# Brain Mass (Energy) Resistant to Hyperglycaemic Oversupply: A Systematic Review

**DOI:** 10.3389/fnins.2021.740502

**Published:** 2021-11-04

**Authors:** Marie Sprengell, Britta Kubera, Achim Peters

**Affiliations:** Center of Brain, Behavior and Metabolism (CBBM), Medical Clinic 1, University of Lübeck, Lübeck, Germany

**Keywords:** brain energy metabolism, brain adenosine triphosphate, brain mass, blood glucose, type 1 diabetes mellitus, Selfish-Brain theory, systematic review

## Abstract

Cerebral energy supply is determined by the energy content of the blood. Accordingly, the brain is undersupplied during hypoglycaemia. Whether or not there is an additional cerebral energy demand that depends upon the energy content of the brain is considered differently in two opposing theoretical approaches. The Selfish-Brain theory postulates that the brain actively demands energy from the body when needed, while long-held theories, the gluco-lipostatic theory and its variants, deny such active brain involvement and view the brain as purely passively supplied. Here we put the competing theories to the test. We conducted a systematic review of a condition in which the rival theories make opposite predictions, i.e., experimental T1DM. The Selfish-Brain theory predicts that induction of experimental type 1 diabetes causes minor mass (energy) changes in the brain as opposed to major glucose changes in the blood. This prediction becomes our hypothesis to be tested here. A total of 608 works were screened by title and abstract, and 64 were analysed in full text. According to strict selection criteria defined in our PROSPERO preannouncement and complying with PRISMA guidelines, 18 studies met all inclusion criteria. Thirteen studies provided sufficient data to test our hypothesis. The 13 evaluable studies (15 experiments) showed that the diabetic groups had blood glucose concentrations that differed from controls by +294 ± 96% (mean ± standard deviation) and brain mass (energy) that differed from controls by −4 ± 13%, such that blood changes were an order of magnitude greater than brain changes (*T* = 11.5, df = 14, *p* < 0.001). This finding confirms not only our hypothesis but also the prediction of the Selfish-Brain theory, while the predictions of the gluco-lipostatic theory and its variants were violated. The current paper completes a three-part series of systematic reviews, the two previous papers deal with a distal and a proximal bottleneck in the cerebral brain supply, i.e., caloric restriction and cerebral artery occlusion. All three papers demonstrate that accurate predictions are only possible if one regards the brain as an organ that regulates its energy concentrations independently and occupies a primary position in a hierarchically organised energy metabolism.

**Systematic Review Registration:** https://www.crd.york.ac.uk/prospero/display_record.php?RecordID=156816, PROSPERO, identifier: CRD42020156816.

## Introduction

**Figure 1:**
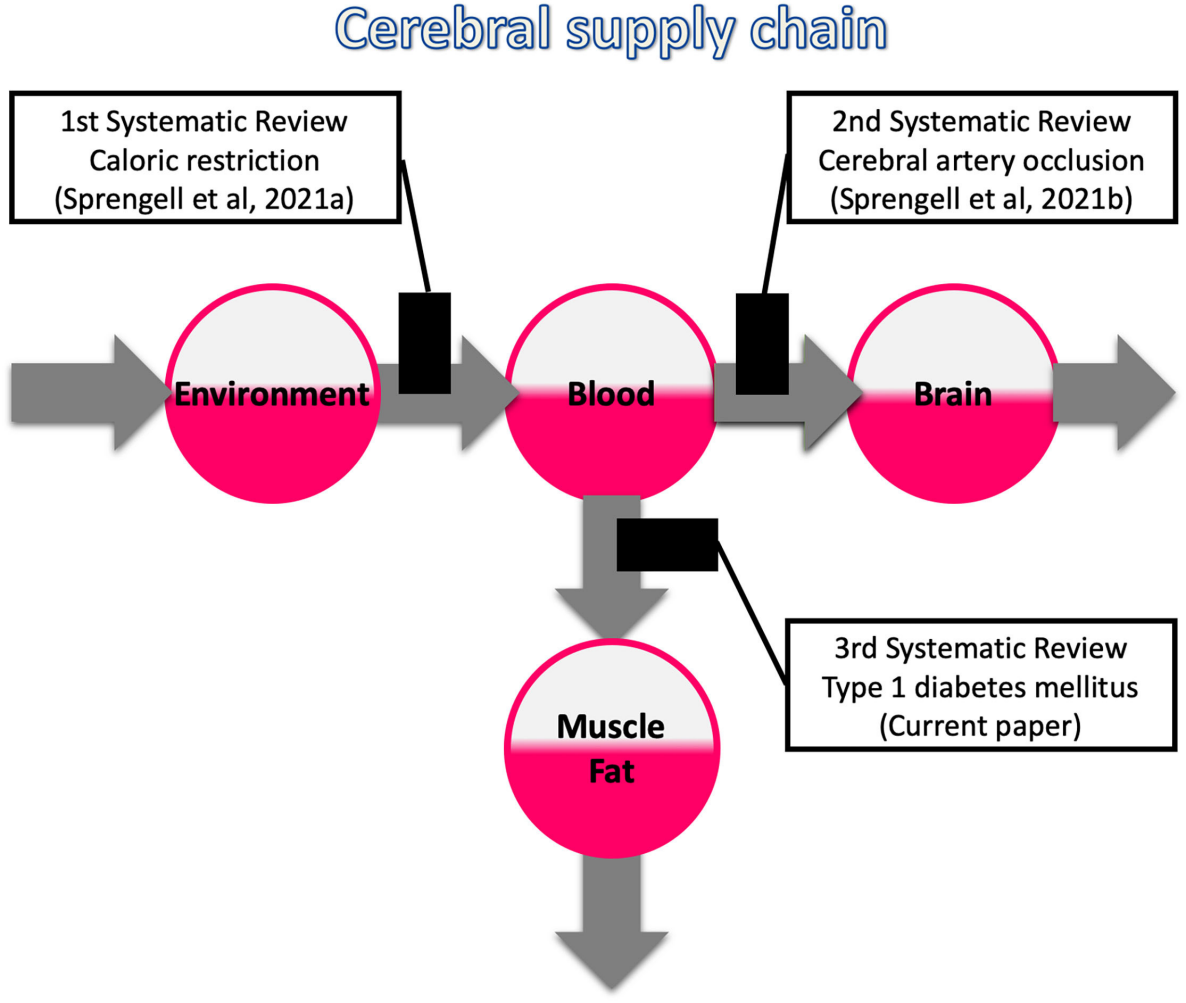
Systematic reviews on the three possible bottlenecks within the cerebral supply chain. The first review deals with a distal bottleneck, i.e., caloric restriction (Sprengell et al., 2021a); the second one, deals with a proximal bottleneck, i.e., cerebral artery occlusion (Sprengell et al., 2021b); the third one, the current work, deals with a peripheral bottleneck, i.e., type 1 diabetes mellitus.

What metabolic abnormalities would you expect when the energy inflow to the blood, brain or muscle/fat tissue is disrupted? This question divides the beliefs. To shed light on this issue, we have completed a three-part series of systematic reviews, the first two of which have recently been published (Sprengell et al., 2021a,b) and the third one we present here with this article (Figure 1).

Disrupted energy inflow to the blood, as occurs with caloric restriction, resulted in minor mass (energy) changes in the brain as opposed to major changes in the body. Disrupted energy inflow into the brain, as occurs with cerebral artery occlusion, led to an increase in systemic blood glucose. Disrupted energy inflow to the muscle/fatty tissue, as it occurs in type 1 diabetes mellitus (T1DM), is subject of the current systematic review.

What is the theoretical background that can lead to controversial predictions? A set of long-held theories on energy metabolism regards the brain as only passively supplied. This is the gluco-lipostatic theory (Kennedy, 1953; Mayer, 1953) and its modern variants (Chaput and Tremblay, 2009; Schwartz et al., 2017). In contrast, the Selfish-Brain theory describes the brain as a self-regulating compartment that primarily covers its own high energy need (Peters et al., 2004). In this respect, the brain behaves “selfishly.” The current systematic review aims to test whether the gluco-lipostatic theory or the Selfish-Brain theory makes more accurate predictions.

The gluco-lipostatic model consists of two components, the lipostatic and the glucostatic. The lipostatic theory was founded by Gordon C. Kennedy (Kennedy, 1953). He believed that body mass was the regulated quantity in energy metabolism. He postulated that a signal from the body energy stores controls food intake. Yet he could not name this signal. Kennedy's lipostatic theory gained momentum in the 1990s when leptin was identified as the signal from adipose tissue that he had suspected (Zhang et al., 1994).

Glucostatic theories include Jean Mayer's original “glucostatic theory” and the “blood-glucose-insulin models” pioneered by Bergman and Cobelli (1980). Jean Mayer was the first to formulate a glucostatic theory (Mayer, 1953). In his model of energy metabolism, blood glucose is the most important physiological variable to be regulated and should be kept as constant as possible. That is why he called his model “glucostatic theory.” The “blood-glucose-insulin models” also see blood glucose as the main regulated variable. While Mayer's model assumed blood glucose to be controlled by adjusting food intake, Cobelli's and Bergman's blood-glucose-insulin models assumed blood glucose to be controlled by adjusting insulin-dependent storage in muscle and fat tissue. Against this background, Mayer's glucostatic model is only one representative of a class of glucostatic theories that consider keeping blood glucose levels constant as the main goal in energy metabolism.

The gluco-lipostatic theory and its modern variants, which combine Kennedy's and Mayer's model with a blood glucose-insulin model, have in common that they see the brain as passively supplied. This view on the brain becomes particularly clear in the blood glucose-insulin models, as they are formulated in mathematically explicit form. Cobelli's blood glucose-insulin model postulated that insulin-independent utilisation is constant and represents glucose uptake by the brain and erythrocytes (Dalla Man et al., 2007; Li et al., 2016). Therefore, in this model, the brain is supplied independently of the cerebral energy content, i.e., in a passive manner. Bergman's so-called “minimal model” postulated that insulin and glucose *per se* influence net glucose utilisation (i.e., production by the liver minus uptake by brain and muscle; Bergman, 2005). The latter assumption means that Bergman also saw the brain as only passively supplied.

**Figure 2:**
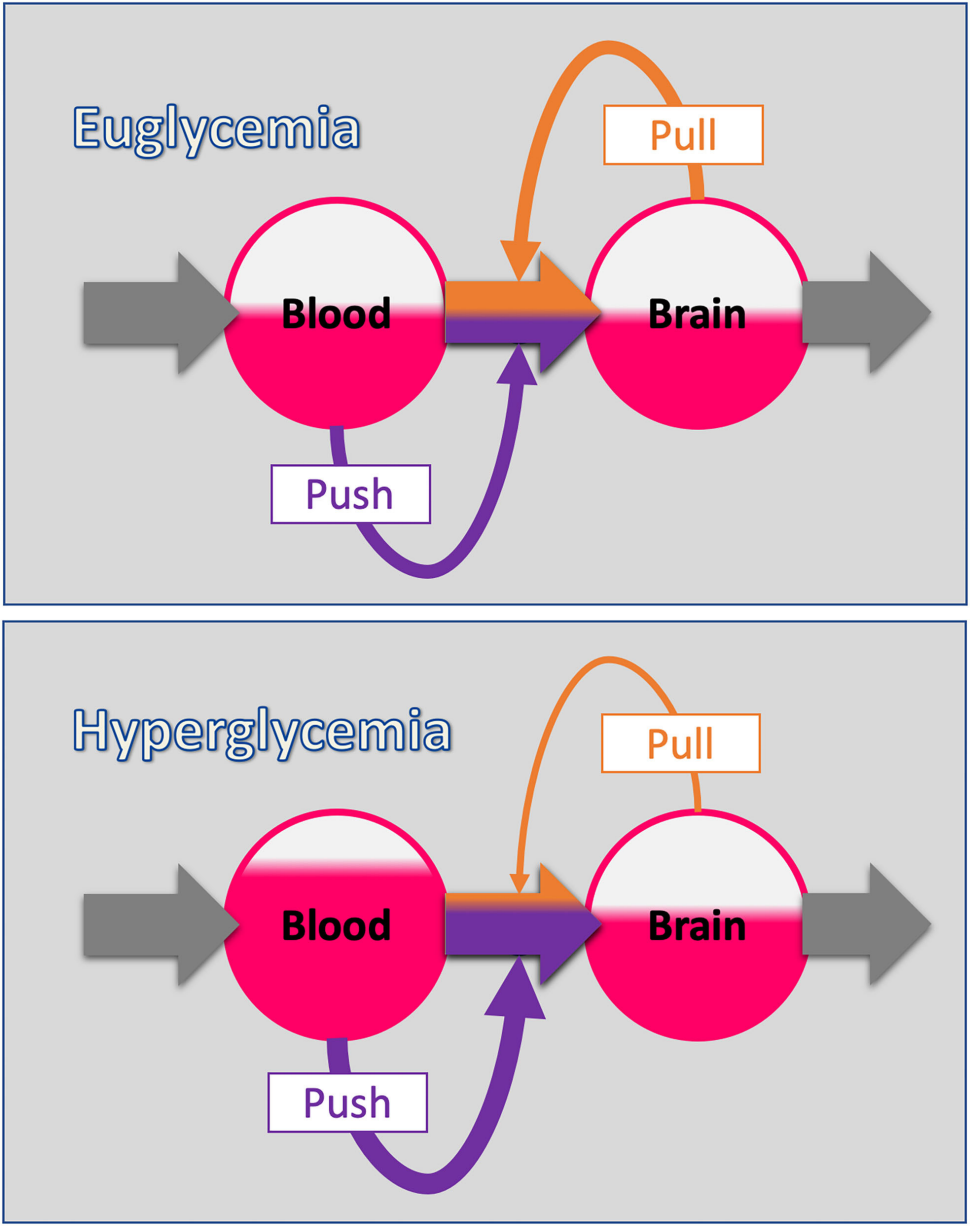
The push-pull principle in the cerebral supply chain. (Upper panel) The blood-to-brain energy flow is determined by two components: The push-component depends on the energy level of the blood (blood glucose). The pull-component depends on the energy level of the brain (intraneuronal ATP). This principle corresponds to the economic concept of “supply and demand.” (Lower panel) When the blood energy content increases (hyperglycaemia), the blood-push component increases and with it the proportion of the blood-to-brain flow determined by the blood (purple share of flow). Note that the energy level in the respective station of the supply chain is indicated by the area coloured red. Oversupply by an increased blood-push leads compensatory to a reduction in the brain-pull component decreasing the proportion of blood-to-brain flow determined by the brain (orange share of flow). In this way, fluctuations in the energy level of the end consumer (the brain in this case) are minimised, which basically is the function of supply chains.

The Selfish-Brain theory is formalised as a supply chain model (Peters and Langemann, 2009). Herein, the brain occupies the position of the final consumer. The brain is the organ that consumes the largest share of glucose in the organism (Reinmuth et al., 1965). In the cerebral supply chain, energy from the environment is taken up by the body (into the blood stream), and from there ~2/3 of the circulating glucose enters the brain. In the field of logistics such supply chains have been extensively studied (Slack et al., 2004). The logistic push-principle operates according to the following rule: the supplier delivers material and in so doing determines the activity of a production step. In contrast, the pull-principle works in the following manner: the material required for a production step is provided only when the receiver needs it (just-in-time). In comparison with the push-principle the pull-principle offers clear economic advantages; with the latter there are short set-up times and only small (economically optimised) storage sites (Figure 2, upper panel).

In the neuroenergetic supply chain, the “brain-pull” refers to the force with which the brain actively demands energy from the body. This brain-pull is the very feature that distinguishes the Selfish-Brain theory from the gluco-lipostatic theory and its variants. The “body-pull” is the force with which the body demands energy from the environment – a pull component that we usually refer to as hunger and which also occurs in the gluco-lipostatic model.

The existence of the postulated brain-pull mechanisms was supported by evidence from the 1990s showing that when a neuron fires and needs more energy to do so, it pulls glucose from the blood via the astrocytes (Pellerin and Magistretti, 1994). Against this background, the brain turns into an active part in energy metabolism according to the principle of “energy on demand” (Magistretti et al., 1999). Other brain-pull mechanisms have been discovered that are activated by sensors in amygdala (Zhou et al., 2010) and ventromedial hypothalamus (VMH) (Spanswick et al., 1997; Routh et al., 2014; Toda et al., 2016) when intraneuronal adenosine triphosphate (ATP) falls. These neuroendocrine mechanisms involve the sympathetic nervous system and the hypothalamic-pituitary-adrenal axis: first, cerebral insulin suppression prevents the body stores to take up glucose so that more glucose is available to the brain (Woods and Porte, 1974; Ahren, 2000; Hitze et al., 2010); second, visceral lipolysis and hepatic ketogenesis increase the availability of ketones to the brain as alternative energy substrates (Kubera et al., 2014); and third, acceleration of the heart rate increases the blood volume that reaches the brain, procuring it with additional energy (Jones et al., 2011).

A brain that works according to the energy-on-demand principle will under certain circumstances produce different metabolic outcomes than its passive counterpart. For a given model of energy metabolism, the expected outcomes will influence individual treatment decisions, guidelines, and practical recommendations, for example, for obesity, type 2 diabetes mellitus, and cardiovascular disease. Since such decisions can have far-reaching consequences for the individual and society, clarification should be brought about by testing the theories at issue.

So far, two out of three systematic reviews have put the theory of the Selfish-Brain to the test. In our first systematic review, the predictions of the Selfish-Brain theory were met in terms of what happens when the energy flow to the blood is disrupted (calorie restriction), while the predictions of the gluco-lipostatic theory and its variants were violated (Sprengell et al., 2021a). As mentioned above, during caloric restriction, the brain mass (energy) was much less affected than that of the body (Sprengell et al., 2021a). From the perspective of the gluco-lipostatic theory and its variants, brain and body should be equally affected by the lack of energy – which was not the case.

In our second systematic review, the Selfish-Brain theory continued to demonstrate the accuracy of its predictions by passing the second test, which dealt with disrupted energy inflow to the brain (occlusion of the cerebral artery) (Sprengell et al., 2021b). As already noted, the occlusion of the cerebral arteries increased systemic blood glucose (Sprengell et al., 2021b). The gluco-lipostatic theory and its variants failed with their prediction that states that during cerebral ischaemia the blood glucose level would remain “static” (i.e., kept constant). After all, this was not the case either.

The aim of our third systematic review, the current paper, is to again test the predictions of the Selfish-Brain theory against those of the gluco-lipostatic theory in experimental T1DM. Therefore, we have formulated a hypothesis on this issue that, if confirmed, would match the predictions of the Selfish-Brain theory – but not those of the gluco-lipostatic theory:

*Hypothesis: Experimental T1DM causes minor mass (energy) changes in the brain as opposed to major glucose changes in the blood*.

To this end, we systematically searched the literature to test whether the T1DM studies we found actually confirm this hypothesis or not.

## Materials and Methods

We have pre-registered the protocol for this systematic review on PROSPERO on 30th of January 2020; updated versions were published on 28th of September 2020, 14th of December 2020, 24th of February 2021, and 9th of April 2021 (International prospective register of systematic reviews; CRD42020156816). The PRISMA (Preferred Reporting Items for Systematic Reviews and Meta-analyses) guidelines for systematic reviews of interventions have been followed (Moher et al., 2009) and the Cochrane Handbook for systematic reviews of interventions was used (Higgins and Thomas, 2019).

### Search Strategies

We conducted a systematic search of the literature to identify mammal studies that focused on how experimental T1DM affects blood glucose concentrations vs. brain mass or energy (ATP). One reviewer developed the search strategies, which were then discussed with the two other reviewers. The databases of MEDLINE and BIOSIS Previews were searched from their inception to 12th of April 2021, using a combination of key words and in case of the first database MeSH terms. The full MEDLINE and BIOSIS search strategies are provided in the Supplementary Material. Briefly, the search strategies included terms relating to the intervention (experimental type 1 diabetes induced by either chemicals, e.g., streptozotocin or alloxan, or pancreatectomy), to the outcome (brain mass or energy) and to the methodical approach (experimental study), combined by the Boolean operator AND. Synonyms for terms were combined with the operator OR.

### Study Selection

**Figure 3:**
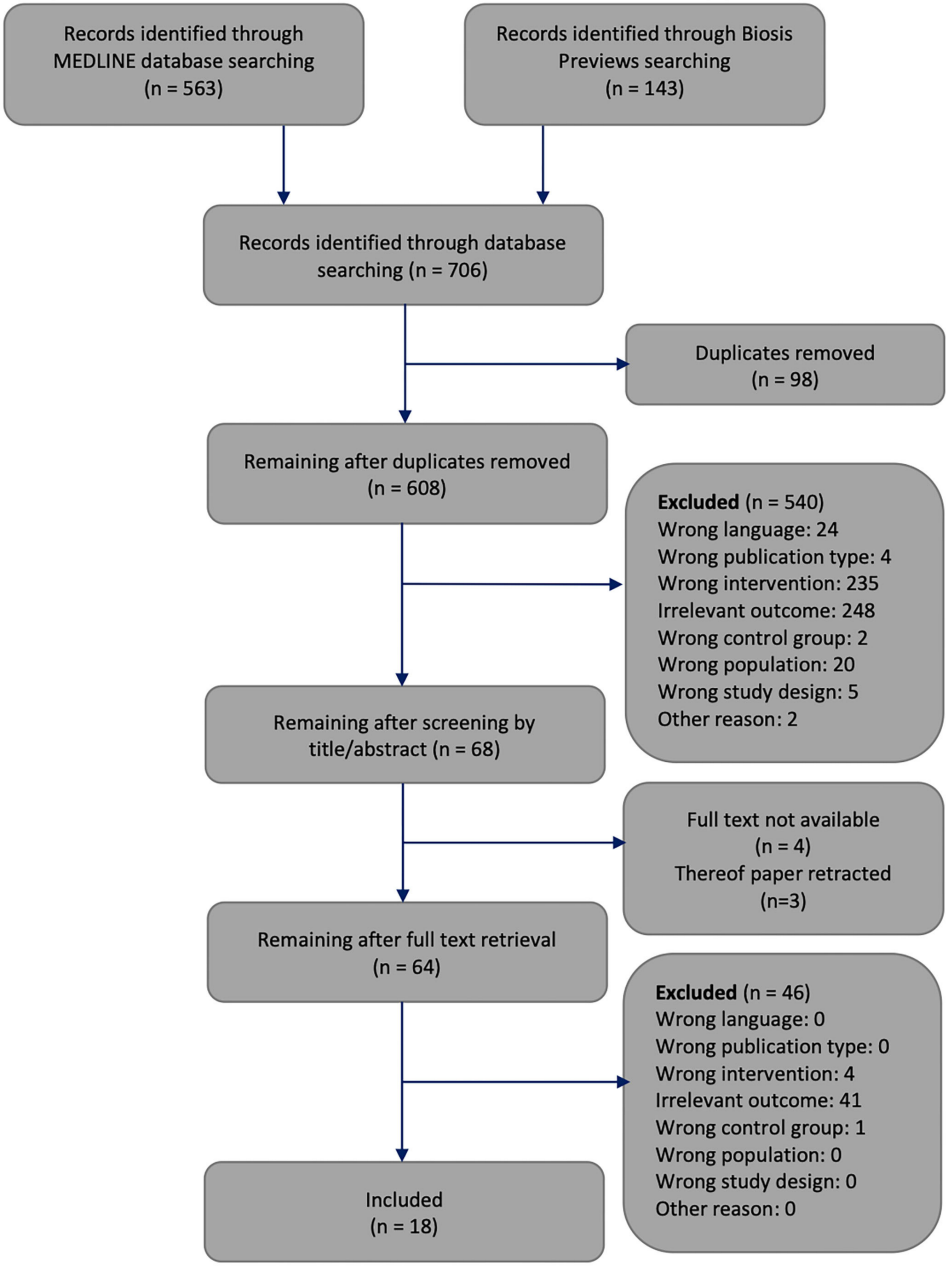
Flowchart through different phases of the systematic review. The framework of this flowchart was taken from Moher's publication (Moher et al., 2009).

The following criteria were used to include or exclude articles for our systematic review. We only included original full research papers published in English or German that examined mammals of any species or sex. We included interventional studies that were standardised laboratory experiments or clinical trials examining two groups, an interventional group in which T1DM was induced and a non-exposed control group. Since we had included clinical trials in our first systematic review (Sprengell et al., 2021a), we did the same here for the sake of consistency, but of course did not expect to identify clinical trials, since the induction of type 1 diabetes in humans is not ethically defensible. We only included studies that provided information about both central (brain ATP or brain mass) and peripheral energy states (blood glucose), since only studies that map both compartments allow us to make comparisons between brain and body. If the brain mass was given as central energy state, the body weight was also demanded. We further only included studies that examined the brain as a whole or large part of the brain, and not just specific regions. We did not include genetical induced diabetes models or neonatal diabetes or trials in pregnant individuals or foetuses, nor in ovariectomised or genetically modified mammals with altered energy metabolism. Moreover, studies, that examined type 2 diabetes mellitus or implemented high-fat diets were excluded. We did not include studies that used combined interventions. We further did not include studies in which the mammals had diseases or were on medication (for more details, see Sprengell et al., 2021a). The selection of items was done in two steps, in each of which we gave the reasons for the exclusion of items (Figure 3). First, one reviewer screened the article titles or abstracts against the inclusion and exclusion criteria. This step of title and abstract screening was checked by another reviewer. Discrepancies regarding the inclusion or exclusion of an article were discussed, remaining disagreements were resolved by consulting the third reviewer. Second, two reviewers independently selected the remaining articles by analysing the full text. Again, disagreements regarding the inclusion or exclusion of an article were resolved by discussion or, if necessary, by consultation with the third reviewer.

### Data Extraction

Data from all 18 included studies were extracted by one reviewer, to be independently checked by the two other reviewers. These studies and the data extracted from them were sorted alphabetically by the name of the first author. We recorded the population, sample size, kind of intervention, statistical test applied, as well as body and brain outcomes.

### Risk of Bias Assessment

To assess the risk of bias of non-human studies the SYRCLE‘s tool was used (Hooijmans et al., 2014). One reviewer assessed the risk of bias of the 18 included studies. The results were independently checked by two other reviewers. All differences were clarified by discussion.

### Statistical Analysis and Hypothesis Decision

Data analysis was performed using SPSS statistical software (SPSS 26.0, Inc., Chicago, USA). We first calculated the percentage changes of brain and body outcomes (compared to controls) from the data shown in Table 1. The Kolmogorov-Smirnov test showed that both the percentage changes in brain outcomes (*p* = 0.163) and the percentage changes in blood glucose concentrations (*p* = 0.200) met the criteria for a normal distribution. Hypothesis decision was made on the basis of paired *t*-test that determined whether percentage changes in blood glucose concentrations (compared to controls) and percentage changes in brain mass (energy) (compared to controls) differed in a statistically significant manner. Based on the statistical test, we decided whether our hypothesis could be confirmed (*p* < 0.05) or not (*p* ≥ 0.05).

**Table 1:** Characteristics and results of included studies.

**References**	**Population**	**Sample size**	**Intervention**	**Statistical test**	**Body outcome**		**Brain outcome**	
					**Blood glucose [mg/dl]**	**Body mass [g]**	**Brain mass [mg]**	**Brain ATP**
Banks et al. (1997)	Mice, male	Exp_1_: 35 Con_1_: 36 Exp_2_: 33 Con_2_:33^a^	Exp_1_: I.p. injection of STZ (5 mg) diluted in vehicle; controls received i.p. injection of vehicle Exp_2_: I.v. injection of alloxan (5 mg) diluted in vehicle into the jugular vein; controls received a jugular injection of vehicle	ANOVA	**Day 2** Exp_1_: 353 ± 149^b^ Con_1_: 299 ± 38^ns^^,^^b^ **Day 5** Exp_1_: 642 ± 105^b^ Con_1_: 266 ± 24^**^^b^ **Day 9** Exp_1_: 782 ± 115^b^ Con_1_: 305 ± 29^**^^b^ **Day 14** Exp_1_: 792 ± 102^b^ Con_1_: 263 ± 38^**^^b^ **Day 2** Exp_2_: 821 ± 133^b^ Con_2_: 314 ± 70^**^^b^ **Day 4** Exp_2_: 732 ± 199^b^ Con_2_: 231 ± 35^**^^b^ **Day 5** Exp_2_: 649 ± 141^b^ Con_2_: 299 ± 56^**^^b^ **Day 7** Exp_2_: 918 ± 110^b^ Con_2_: 325 ± 42^**^^b^	**Day 2** Exp_1_: 31.2 ± 2.2^b^ Con_1_.: 32.1 ± 2.2^ns^^,^^b^ **Day 5** Exp_1_: 20.1 ± 3.6^b^ Con_1_.: 27.6 ± 0.9^**^^b^ **Day 9** Exp_1_: 23.4 ± 3.2^b^ Con_1_.: 28.5 ± 2.4^**^^b^ **Day 14** Exp_1_: 24.1 ± 2.7^b^ Con_1_.: 31.0 ± 2.2^**^^b^ **Day 2** Exp_2_: 26.3 ± 1.3^b^ Con_2_: 27.5 ± 1.3^ns^^,^^b^ **Day 4** Exp_2_: 36.8 ± 2.5^b^ Con_2_: 38.3 ± 3.5^ns^^,^^b^ **Day 5** Exp_2_: 25.2 ± 0.9^b^ Con_2_: 31.3 ± 1.6^**^^b^ **Day 7** Exp_2_: 28.6 ± 2.0^b^ Con_2_: 33.6 ± 2.3^**^^b^	**Day 2** Exp_1_: 457 ± 22^b^ Con_1_: 467 ± 16^ns^^,^^b^ **Day 5** Exp_1_: 450 ± 13^b^ Con_1_: 472 ± 13^**^^b^ **Day 9** Exp_1_: ND Con_1_: ND **Day 14** Exp_1_: 458 ± 15^b^ Con_1_: 485 ± 19^**^^b^ **Day 2** Exp_2_: 479 ± 16^b^ Con._2_: 488 ± 25^ns^^,^^b^ **Day 4** Exp_2_: 491 ± 19^b^ Con._2_: 487 ± 25^ns^^,^^b^ **Day 5** Exp_2_: 443 ± 22^b^ Con._2_: 453 ± 22^ns^^,^^b^ **Day 7** Exp_2_: 478 ± 23^b^ Con._2_: 493 ± 26^ns^^,^^b^	
Biessels et al. (2001)	Wistar rats, 3-month-old, male	Exp: 7 Con: 8	I.v. injection of STZ (30 mg/kg body mass) dissolved in saline^c^	Mann-Whitney *U*-test (P-MRS), *t*-test (body mass, glucose)	**Baseline** Exp: 106 ± 14^b^^,^^d^ Con: 99 ± 10^b^^,^^d^^,^^e^ **Month 8** Exp: 470 ± 67^b^^,^^d^ Con: 124 ± 10^*^^b^^,^^d^^,^^f^	**Baseline** Exp: 353 ± 21^b^ Con: 356 ± 14^b^^,^^e^ **Month 8** Exp: 298 ± 45^b^ Con: 649 ± 61^*^^b^^,^^f^		**Month 8** Exp: 3.01 (2.5–3.4)^g^ Con: 2.95 (2.4–3.9)^ns^^,^^f^^,^^g^ (ATP/Pi)
Cardoso et al. (2010)	Wistar rats, 3-month-old, male	Exp: 12–14 Con: 12–14	I.p. injection of STZ (50 mg/kg body mass) dissolved in citrate; controls received an i.p. injection of citrate; BG > 250 mg/dl was classified as diabetic	Mann-Whitney *U*-test	**Month 1** Exp: 415 ± 94^b^^,^^h^ Con: 89 ± 11^***^^b^^,^^h^	**Month 1** Exp: 259 ± 40^b^^,^^h^ Con: 354 ± 14^***^^b^^,^^h^	**Month 1** Exp: 2,400 ± 360^b^^,^^h^ Con: 2,500 ± 720^ns^^,^^b^^,^^h^	
Cardoso et al. (2013a)	Wistar rats, 2-month-old, male	Exp: 6 Con: 6	I.p. injection of STZ (50 mg/kg body mass) dissolved in citrate; controls received i.p. injection of citrate; BG > 250 mg/dl was classified as diabetic	Mann-Whitney *U*-test	**Month 3** Exp: 453 ± 54^b^ Con: 127 ± 14^***^^b^	**Month 3** Exp: 296 ± 21^b^ Con: 440 ± 15^***^^b^	**Month 3** Exp: 2,100 ± 170^b^ Con: 2,050 ± 70^ns^^,^^b^	
Cardoso et al. (2013b)	Wistar rats, 2-month-old, male	Exp: 6–8 Con: 6–8	I.p. injection of STZ (50 mg/kg body mass) dissolved in citrate; controls received i.p. injection of citrate; BG > 250 mg/dl was classified as diabetic	Mann-Whitney *U*-test	**Month 3** Exp: 467 ± 50^b^^,^^i^ Con: 123 ± 13^***^^b^^,^^i^	**Month 3** Exp: 300 ± 21^b^^,^^i^ Con: 444 ± 14^***^^b^^,^^i^	**Month 3** Exp: 2,130 ± 190^b^^,^^i^ Con: 2,060 ± 80^ns^^,^^b^^,^^i^	
Cintra et al. (2017)	Wistar rats, male	Exp: 10 Con: 8	I.v. injection of STZ dissolved in citrate buffer; controls received i.v. injection of citrate buffer; BG > 200 mg/dl was classified as diabetic	Two-way ANOVA followed by the Holm-Sidak method for pairwise multiple comparisons	**Day 0** Exp: 76 ± 12 Con: 81 ± 11^ns^ **Day 6** Exp: 384 ± 124 Con: 107 ± 13^*^^f^	**Day 0** Exp: 250^j^^,^^k^ Con: 250^ns^^,^^j^^,^^k^ **Day 6** Exp: 274^j^^,^^k^ Con: 305^ns^^,^^f^^,^^j^^,^^k^	- ^l^	
Jakobsen et al. (1987)	Wistar rats, 22-week-old, male	Exp: 5 Con: 4	I.v. injection of STZ (40 mg/kg body mass); start of insulin treatment (1 IU/day) after 25 weeks; BG > 250 mg/dl was classified as diabetic	Students unpaired *t*-test	**Baseline** Exp: 105^d^^,^^k^^,^^m^ Con: not given^e^ **Week 59** Exp: 375 ± 33^d^^,^^k^ Con: not given^e^^,^^f^^,^^k^	**Baseline** Exp: 393 ± 17^b^^,^^k^ Con: 405 ± 15^b^^,^^e^^,^^k^ **Week 59** Exp: 380 ± 17^b^^,^^k^ Con: 463 ± 70^b^^,^^e^^,^^f^^,^^k^	**Week 59** Exp: 1,350 ± 70 Con: 1,520 ± 60^**^^n^	
Katyare and Patel (2006)	Albino Wistar rats, 190–230 g initial body mass, male and female	Exp(m): 8 Exp(f): 10 Con(m): 9 Con(f): 10	I.p. injection of STZ (65 mg/kg body mass) dissolved in citrate buffer; controls received injection of vehicle	*t*-test	**Month 1** Exp(m): 450 ± 76 Con(m): 121 ± 12^***^ Exp(f): 485 ± 136 Con(f): 110 ± 16^***^	**Month 1** Exp (m): 236.3 ± 47.0 Con(m): 417.0 ± 49.2^***^ Exp(f): 218.5 ± 18.7 Con(f): 268.5 ± 16.1^***^	**Month 1** Exp(m): 2,060 ± 170 Con(m): 2,180 ± 120^ns^ Exp(f): 1,970 ± 30 Con(f): 1,860 ± 90^***^	
Mans et al. (1988)	Long-Evans rats, 255–275 g initial body mass, male	Exp: 8^o^ Con: 7	I.v. injection of STZ (65 mg/kg body mass) dissolved in citrate buffer^p^	ANOVA with the Bonferroni correction	Con: 133 ± 34^b^^,^^d^^,^^q^ **Week 1** Exp: 534 ± 93^*^^b^^,^^d^ **Week 4** Exp: 293 ± 57^*^^b^^,^^d^			Con: 2.83 ± 0.11^b^^,^^q^ **Week 1** Exp: 2.89 ± 0.13^ns^^,^^b^ **Week 4** Exp: 2.78 ± 0.12^ns^^,^^b^^,^^r^ (μmol/g wet mass)
Marissal-Arvy et al. (2018)	Sprague Dawley rats, 3-week-old, male	Exp.: 6 Con.: 5	I.p. injection of STZ (65 mg/kg body mass per day for 2 days); controls received i.p. injection of citrate buffer; BG > 200 mg/dl was classified as diabetic	One-way ANOVA followed by Fisher LSD *post-hoc* test	Exp. vs. Con^***^ (Data not shown)	Exp. vs. Con^***^ (Data not shown)	**Week 3** Exp: 510 ± 50^b^^,^^k^ Con: 600 ± 50^*^^b^^,^^k^ (volume in mm^3^)	
Mastrocola et al. (2005)	Wistar rats, male	Exp.: 6–7 Con: 6–7	I.v. injection of STZ (50 mg/kg body mass); BG of 324 mg/dl to 360 mg/dl was classified as diabetic and entered protocol^s^	Students *t*-test	**Day 21** Exp: 373 ± 41^d^^,^^e^ Con:109 ± 12^d^^,^^e^	**Day 21** Exp: 224.7 ± 10.4 Con: 250.9 ± 12.4^*^		**Day 21** Exp: 23 ± 2^k^ Con: 29 ± 3^*^^k^^,^^t^ (μmol/l)
Min et al. (2020)	ICR mice (albino mice), 5-week-old, male	Exp: 10 Con: 10	I.v. injection of alloxan solution (50 mg/kg body mass); BG > 240 mg/dl was classified as diabetic	No comparison between alloxan and control group	**Week 12** Exp: 472 ± 120^k^ Con: 120^e^^,^^f^^,^^j^^,^^k^	**Week 12** Exp: 30 ± 3^k^ Con: 41 ± 3^e^^,^^f^^,^^k^	-^l^^,^^u^	
Moreira et al. (2004)	Wistar rats, 3-month-old, male	Exp: 8 Con: 8	I.p. injection of STZ (50 mg/kg body mass) dissolved in citrate; controls received injection of citrate; BG > 250 mg/dl was classified as diabetic	One-way analysis of variance for multiple comparisons, followed by the *post-hoc* Tukey-Kramer test	**Week 4** Exp: 412 ± 52^b^^,^^v^ Con: 118 ± 17^***^^b^^,^^v^ **Week 9** Exp: 458 ± 59^b^^,^^v^ Con: 100 ± 11^***^^b^^,^^v^	**Week 4** Exp: 89.7 ± 10.5%^b^ Con: 123.6 ± 30.3%^***^^b^ **Week 9** Exp: 87.8 ± 15.8%^b^ Con: 190.5 ± 25.5%^***^^b^ (percentage of initial mass)		**Week 4** Exp: 109 ± 12.0%^b^ Con: 100 ± 18.5%^ns^^,^^b^ **Week 9** Exp: 101 ± 18.4%^b^ Con: 85.8 ± 16.9%^ns^^,^^b^^,^^w^ (percentage of 4-week control rats)
Moreira et al. (2005)	Wistar rats, 10-week-old, male	Exp: 6 Con: 6	I.p. injection of STZ (50 mg/kg body mass) dissolved in citrate; controls received injection of citrate; BG > 250 mg/dl was classified as diabetic	Students *t*-test	**Week 12** Exp: 527 ± 61^b^^,^^v^ Con: 93 ± 9^***^^b^^,^^v^	**Week 12** Exp: 331.8 ± 52.9^b^ Con: 517.8 ± 19.8^***^^b^		**Week 12** Exp: 128 ± 6^b^ Con: 153 ± 7^*^^b^^,^^w^ (mmol/mg protein)
Moreira et al. (2006)	Wistar rats, 12-week-old, male	Exp: 5 Con: 5	I.p. injection of STZ (50 mg/kg body mass) dissolved in citrate; controls received injection of citrate; BG > 250 mg/dl was classified as diabetic	Student *t*-test	**Week 12** Exp: 527 ± 56^b^^,^^v^ Con: 93 ± 9^***^^b^^,^^v^	**Week 12** Exp: 331.8 ± 48.3^b^ Con: 517.8 ± 18.1^***^^b^	**Week 12** Exp: 1300 ± 720^b^ Con: 1980 ± 130^*^^b^^,^^w^	
Thurston et al. (1975)	White mice, 18–23 days old	Exp: 17–23^x^ Con: 17–23^x^	I.v. injection of alloxan (100 mg/kg body mass) dissolved in NaCl; BG > 250 mg/dl was classified as diabetic	Unclear	**Day 4** Exp: 685 ± 267^b^^,^^d^ Con: 166 ± 24^***^^b^^,^^d^	**Baseline** Exp and Con: 8.6 ± 1.2^b^ **Day 4** Exp: 7.3 ± 1.4^b^ Con: 12.7 ± 2.2^e^		**Day 4** Exp: 2.98 ± 0.24^b^ Con: 2.80 ± 0.10^**^^b^ (mol/kg)
Tomassoni et al. (2004)	Wistar-Kyoto rats, 10-week-old	Exp: 6 Con: 6	I.p. injection of STZ (50 mg/kg body mass) dissolved in citrate buffer; controls received i.p. injection of citrate buffer; BG > 300 mg/dl was classified as diabetic^y^	ANOVA followed by the Newmann-Keuls multiple range test	**Week 8** Exp.: 470 ± 24^b^ Con.: 200 ± 12^*^^b^	**Week 8** Exp: 221 ± 34.3 Con: 336 ± 24.5^*^^b^	**Week 8** Quote: “No differences in brain mass…” (data not shown)	
Zhou et al. (2018)	Wistar rats, 8–10-week-old, male	Exp: 45^z^ Con: 12^aa^	I.p. injection of STZ (55 mg/kg body mass) dissolved in citrate-sodium citrate buffer; controls received injection of citric acid-sodium citrate buffer; BG > 301 mg/dl was classified as diabetic	One-way ANOVA	**Day 63** Exp: 432 ± 180^d^^,^^k^ Con: 117 ± 27^**^^d^^,^^f^^,^^k^	**Baseline** Exp.: 295 ± 20^k^ Con.: 287 ± 18^ns^^,^^k^ **Day 63** Exp: 275 ± 25^k^ Con: 450 ± 40^**^^f^^,^^k^	**Day 90**^bb^ Exp_cog_: 3.31 ± 0.28^*^^cc^ Exp_non−cog_: 3.27 ± 0.22^*^^cc^ Con: 3.61 ± 0.26 (volume in cm^3^)	

*Mean and standard deviation are presented. Values are given in the units listed in the first row of the table, unless otherwise stated. The values from the original papers were rounded if there were too many decimal places. i.p., intraperitoneal; STZ, streptozotocin; i.v., intravenous; ND, not determined; ANOVA, analysis of variance; h, hour; MRI, magnetic resonance imaging; ns, not significant; P-MRS, phosphate magnetic resonance spectroscopy; S.E.M., standard error of the mean*;

* *p < 0.05*,

** *p < 0.01*,

*** *p < 0.001*.

a *At the time of each measurement about 10 animals were killed and data were collected*.

b *S.E.M. was provided in the original paper. We converted the S.E.M. to standard deviation using the following formula: Standard deviation = S.E.M. × $\sqrt{sample\;size}$*.

c *Fluanisone, fentanylcitrate, and midazolam anaesthesia for MRS procedure*.

d *Blood glucose was presented in [mmol/l] (or [μmol/ml]) in the original paper and converted by the present reviewer to [mg/dl] with the conversion factor 18.02*.

e *No data on statistical testing*.

f *More data available. Only pairs of body outcome and brain outcome measured at the same time are shown*.

g *Median (interquartile range)*.

h *Sample size is stated with “12–14 animals”; to calculate the standard deviation, we assumed 13 animals*.

i *Sample size is stated with “6–8 animals”; to calculate the standard deviation, we assumed 7 animals*.

j *SEM or SD not given*.

k *Value taken from graph; thus no decimal place is presented*.

l *Absolute brain mass cannot be extracted directly, but must be calculated from relative brain mass and body mass, whereby the latter values can only be taken from graphics. This leads to cumulative reading errors. Furthermore, the standard deviation cannot be extracted with this procedure*.

m *SD not readable*.

n *The brain above the mesencephalon was analysed excluding cerebellum and olfactory tissue*.

o *For the determination of brain metabolites, the group size was 1 animal smaller than for the determination of the other outcomes*.

p *Ketamine anaesthesia before sacrificing*.

q *Time of measurement unclear*.

r *All parts of the brain were analysed except for pons, medulla oblongata, cerebellum, and osmic bulbs*.

s *Rats were anaesthetised with ether before killing*.

t *The cerebral hemispheres were analysed*.

u *In the coordinate cross of the relative brain weight plot, the labelling of the ordinate is ambiguous, which makes unambiguous data extraction impossible*.

v *From the order of magnitude it can be assumed that the unit [mg/ml] given in the paper is incorrect; we proceed on the assumption that the authors meant [mg/dl]*.

w *All brain parts were analysed except for the cerebellum*.

x *Litters of various sample sizes were used for different measurements*.

y *Diethyl ether anaesthesia prior to blood glucose measurement*.

z *Out of 73 rats that received STZ*.

aa *To assess the brain mass only 7 + 9 (experimental group) and 7 (control group) animals were examined*.

bb *This point of time is estimated; time of brain volume measurement not explicitly mentioned*.

cc *The experimental group was divided according to cognitive impairment*.

## Results

The systematic search of the literature generated 706 articles, which were processed as summarised in Figure 3. Six hundred and eight works were screened by title or abstract, and 64 articles were analysed by full text. We identified 18 studies that met all inclusion criteria, had no exclusion criteria, and focused on how experimental T1DM affects brain mass or energy vs. blood glucose concentrations.

### Data Extraction

Table 1 provides details of the 18 included studies. Fifteen studies investigated rats (Jakobsen et al., 1987; Mans et al., 1988; Biessels et al., 2001; Moreira et al., 2004, 2005, 2006; Tomassoni et al., 2004; Mastrocola et al., 2005; Katyare and Patel, 2006; Cardoso et al., 2010, 2013a,b; Cintra et al., 2017; Marissal-Arvy et al., 2018; Zhou et al., 2018) and three studies investigated mice (Thurston et al., 1975; Banks et al., 1997; Min et al., 2020). The sample sizes varied between 9 (Jakobsen et al., 1987) and 71 per experiment (Banks et al., 1997).

All 18 included studies provided details on how experimental type 1 diabetes was induced. By far the largest proportion of studies used an injection of streptozotocin (STZ) to induce T1DM (Jakobsen et al., 1987; Mans et al., 1988; Banks et al., 1997; Biessels et al., 2001; Moreira et al., 2004, 2005, 2006; Tomassoni et al., 2004; Katyare and Patel, 2006; Cardoso et al., 2010, 2013a,b; Cintra et al., 2017; Marissal-Arvy et al., 2018; Zhou et al., 2018); in three studies diabetes was induced by alloxan (Thurston et al., 1975; Banks et al., 1997; Min et al., 2020); none of the included studies used pancreatectomy for diabetes induction.

All included studies measured blood glucose concentrations. Nine studies measured brain mass or volume and reported it adequately (Jakobsen et al., 1987; Banks et al., 1997; Katyare and Patel, 2006; Moreira et al., 2006; Cardoso et al., 2010, 2013a,b; Marissal-Arvy et al., 2018; Zhou et al., 2018) and six studies measured brain ATP (Thurston et al., 1975; Mans et al., 1988; Biessels et al., 2001; Moreira et al., 2004, 2005; Mastrocola et al., 2005). Five studies provided incomplete or non-extractable data on either blood glucose concentrations (Jakobsen et al., 1987; Marissal-Arvy et al., 2018) or brain outcomes (Tomassoni et al., 2004; Cintra et al., 2017; Min et al., 2020). In summary, of the 18 studies included 13 were evaluable (Thurston et al., 1975; Mans et al., 1988; Banks et al., 1997; Biessels et al., 2001; Moreira et al., 2004, 2005, 2006; Mastrocola et al., 2005; Katyare and Patel, 2006; Cardoso et al., 2010, 2013a,b; Zhou et al., 2018). Of the 13 evaluable studies, two studies conducted two experiments each (Banks et al., 1997; Katyare and Patel, 2006), so that we ended up with 13 evaluable studies (15 experiments) for our hypothesis decision.

### Risk of Bias Assessment

Table 2 provides the risk of bias assessments of all 18 included studies.

**Table 2:** Risk of bias assessment.

	**Random sequence generation**	**Baseline characteristics**	**Addressing of Incomplete outcome data**	**Selective outcome reporting**	**Other sources of bias**
Banks et al. (1997)	+	?^a^	+^b^	+^c^	+^d^
Biessels et al. (2001)	?	+	+^e^	+	+^d^
Cardoso et al. (2010)	+	?^a^	–^f^	+	+^d^
Cardoso et al. (2013a)	+	?^a^	+^b^	+	+^d^
Cardoso et al. (2013b)	+	?^a^	–^f^	+	+^d^
Cintra et al. (2017)	+	+	+^b^	+	+^d^
Jakobsen et al. (1987)	+	+	+^b^	–^g^	+^d^
Katyare and Patel (2006)	?	?^a^	+^b^	+	+^d^
Mans et al. (1988)	?	?^a^	+^b^	+	-^h^
Marissal-Arvy et al. (2018)	?	?^a^	+^b^	–^i^	?^j^
Mastrocola et al. (2005)	?	?^a^	–^f^	+	+^d^
Min et al. (2020)	?	+	+^b^	?^k^	+^d^
Moreira et al. (2004)	+	?^a^	+^b^	+	+^d^
Moreira et al. (2005)	+	?^a^	+^b^	+	+^d^
Moreira et al. (2006)	+	?^a^	+^b^	+	+^d^
Thurston et al. (1975)	?	?^a^	+^b^	–^l^	+^d^
Tomassoni et al. (2004)	?	?^a^	+^b^	–^m^	+^d^
Zhou et al. (2018)	+	+	–^n^	+	+^o^

*The SYRCLE‘s tool (Hooijmans et al., 2014) was used and slightly modified to assess the risk of bias of non-human studies. “+” indicates a low risk of bias; “–” indicates a high risk of bias; “?” indicates that not enough information has been provided on this item. For five items of the SRYCLE tool, i.e., allocation concealment, random housing, blinding of personnel, random outcome assessment, and blinding of the outcome assessors, not enough information was available for any of the studies examined here, so that all of them were rated “?.”*

a *Baseline values not given*.

b *No evidence of dropouts*.

c *Unlike the other experiments in this list, Bank's experiment reports detailed time courses; as an exception the brain weight values from day 9 are missing*.

d *No evidence of critical housing conditions, problems associated with study design, or conflicts of interest*.

e *One diabetic and one control rat died; Quote: “Spare animals replaced these rats in the final measurements.”*

f *Range of sample size given, so it remains unclear whether there were dropouts*.

g *Blood glucose of control group not given*.

h *Time of measurement of control group unclear; moreover, it remains unclear whether control group underwent sham treatment*.

i *Values of blood glucose and body weight not given*.

j *The research has been supported, inter alia, by Aide aux Jeunes Diabetiques; authors claimed that the funders had no part in any of their work. One author had been paid by Eli Lilly and Novo Nordisk*.

k *In the coordinate cross of the relative brain weight plot, the labelling of the ordinate is ambiguous, making the extraction of this data uncertain*.

l *Only one baseline body weight value is given for both experimental and control group*.

m *Data of brain outcome not shown*.

n *Attrition bias was present as 14 out of 73 rats died in the experimental group; it is appreciated that the number of deceased animals at the different points was presented*.

o *Time of brain volume measurement not explicitly mentioned. No evidence of critical housing conditions, other problems associated with study design, or conflicts of interest*.

### Hypothesis Decision

The 13 evaluable studies (15 experiments) provided data to calculate the percentage changes of brain and body outcomes (compared to controls) (Thurston et al., 1975; Mans et al., 1988; Banks et al., 1997; Biessels et al., 2001; Moreira et al., 2004, 2005, 2006; Mastrocola et al., 2005; Katyare and Patel, 2006; Cardoso et al., 2010, 2013a,b; Zhou et al., 2018).

**Figure 4:**
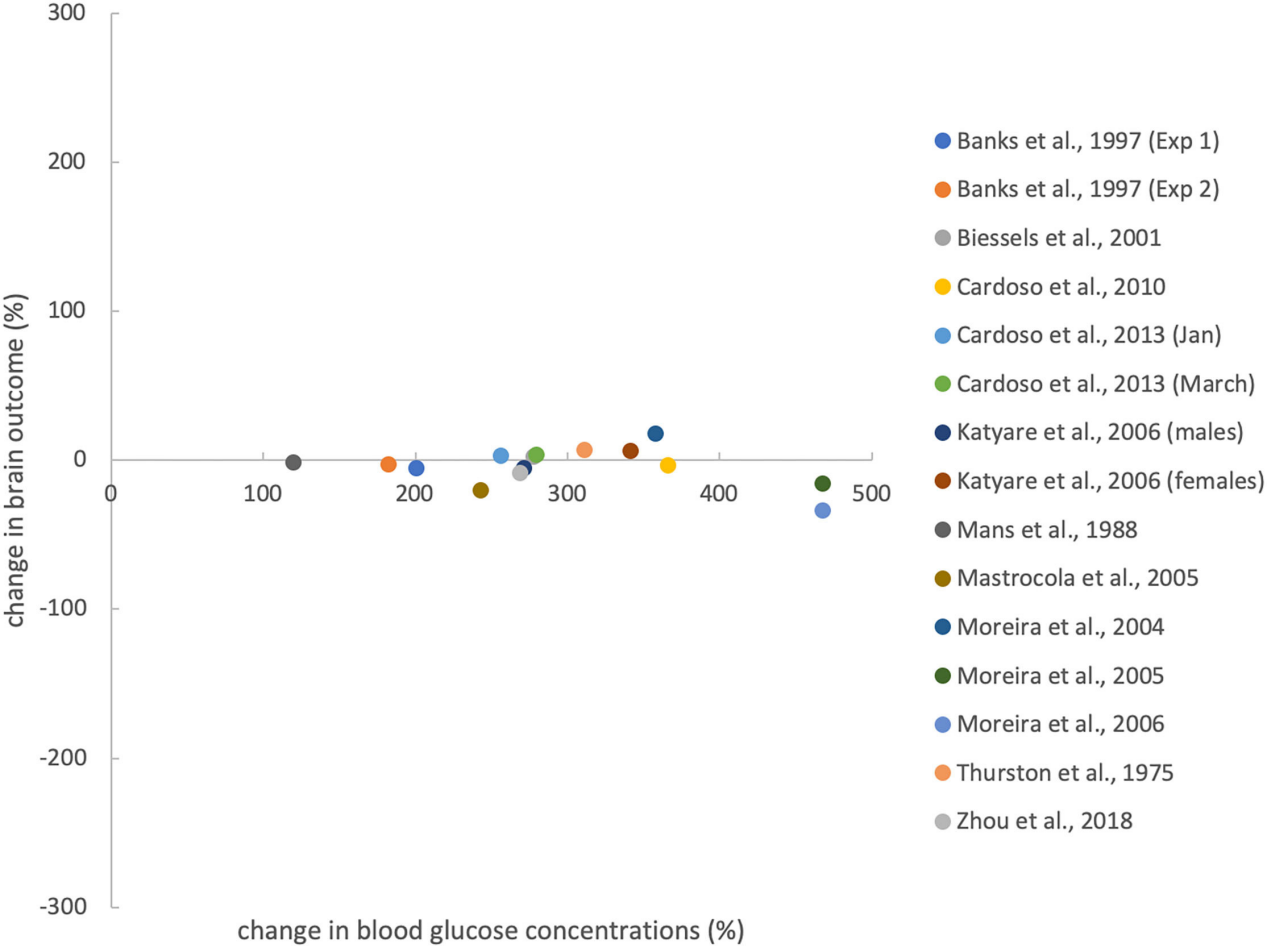
Effects of T1DM induction on percentage change in blood glucose concentration (compared with controls) and brain outcomes. Brain outcomes include brain mass and brain ATP concentrations. Percentage changes were calculated from the data shown in Table 1. For evaluating Zhou et al.'s work, we pooled brain volume data from 2 groups that differed in cognitive function. Anaesthesia before ATP measurement does not confound the relationship between blood and brain outcomes (see Supplementary Material C).

Figure 4 shows that induction of T1DM results in very large percentage changes in blood glucose levels (compared to controls), but only small percentage changes in brain outcomes (compared to controls). The 13 evaluable studies (15 experiments) showed that the diabetic groups had blood glucose concentrations that differed from controls by +294 ± 96% (mean ± standard deviation) and brain mass (energy) that differed from controls by −4 ± 13%, such that blood changes were an order of magnitude greater than brain changes (*T* = 11.5, df = 14, *p* < 0.001; paired *t*-test). In all, our hypothesis that experimental T1DM causes minor mass (energy) changes in the brain, as opposed to major glucose changes in the blood, could be fully confirmed.

## Discussion

A total of 608 works was screened by title and abstract, and 64 were analysed in full text. According to strict selection criteria defined in our PROSPERO pre-announcement and complying with PRISMA guidelines, 18 studies met all inclusion criteria. Thirteen studies provided sufficient data to test our hypothesis. These 13 evaluable studies (15 experiments) showed that in experimental T1DM, major glycaemic disturbance does not translate into major disturbance of brain energy homeostasis. The use of STZ or alloxan to destroy pancreatic beta cells was found to be very effective in producing hyperglycaemia (+294% change compared to controls), but much less effective in affecting brain mass or energy (−4% change compared to controls), leaving the blood compartment much more susceptible to perturbations than the brain (*p* < 0.001) (Figure 4). Thus, all 13 evaluable studies confirmed and none contradicted our hypothesis that experimental induction of T1DM causes minor changes in brain mass (energy) as opposed to major changes in blood glucose levels.

The insight that brain mass (energy) is resistant to hyperglycaemic oversupply is new. Although data on this topic have been available for many years, it is only the synopsis of data and the theoretical background that make the insight possible. Given that the Selfish-Brain theory makes accurate predictions in cases where those of conventional theories have failed (Sprengell et al., 2021a,b), there is reason to believe that the astrocytic-neuronal and systemic-neuroendocrine mechanisms as assigned by the Selfish-Brain theory provide a reliable explanation for brain mass (energy) being resistant to hyperglycaemic oversupply (Peters et al., 2004, 2007, 2017; Peters and McEwen, 2015).

In terms of mechanisms, the Selfish-Brain theory refers to the principle of supply chains stating that “when push increases, pull relaxes” (Figure 2, lower panel). The cerebral-supply-chain model predicts that an increase in the blood-push component (e.g., hyperglycaemia) is compensated by down-regulation of brain-pull components.

Brain-pull function is carried out at the astrocytic level (glutamatergic neuron demands energy from blood) and at the systemic level (brain demands energy from body). The astrocytic brain-pull describes the force with which glutamatergic neurons, when they fire, demand the required energy *via* the astrocytes from the blood (Peters and Langemann, 2009). Pierre Magistretti and Luc Pellerin were the first to describe a brain-pull mechanism and called it the Astrocyte-Neuron Lactate Shuttle (ANLS) (Pellerin and Magistretti, 1994). Since its introduction, the ANLS has received a great deal of attention, with approval as well as criticism; however, substantial evidence has accumulated from independent sources that supports the model (Mason, 2017). The ANLS hypothesis states that firing glutamatergic neurons release glutamate into the synaptic cleft, from where it is taken up by astrocytes; the incoming glutamate is a trigger for astrocytes to take up more glucose, which in turn is converted to lactate to be released *via* monocarboxylate transporters for neuronal use. Thus, according to the ANLS hypothesis, brain-pull activity depends on neuronal glutamatergic activity and subsequent uptake of glutamate in astrocytes. In contrast, GABA does not couple inhibitory neuronal activity with glucose utilisation, as does glutamate for excitatory neurotransmission (Chatton et al., 2003).

**Figure 5:**
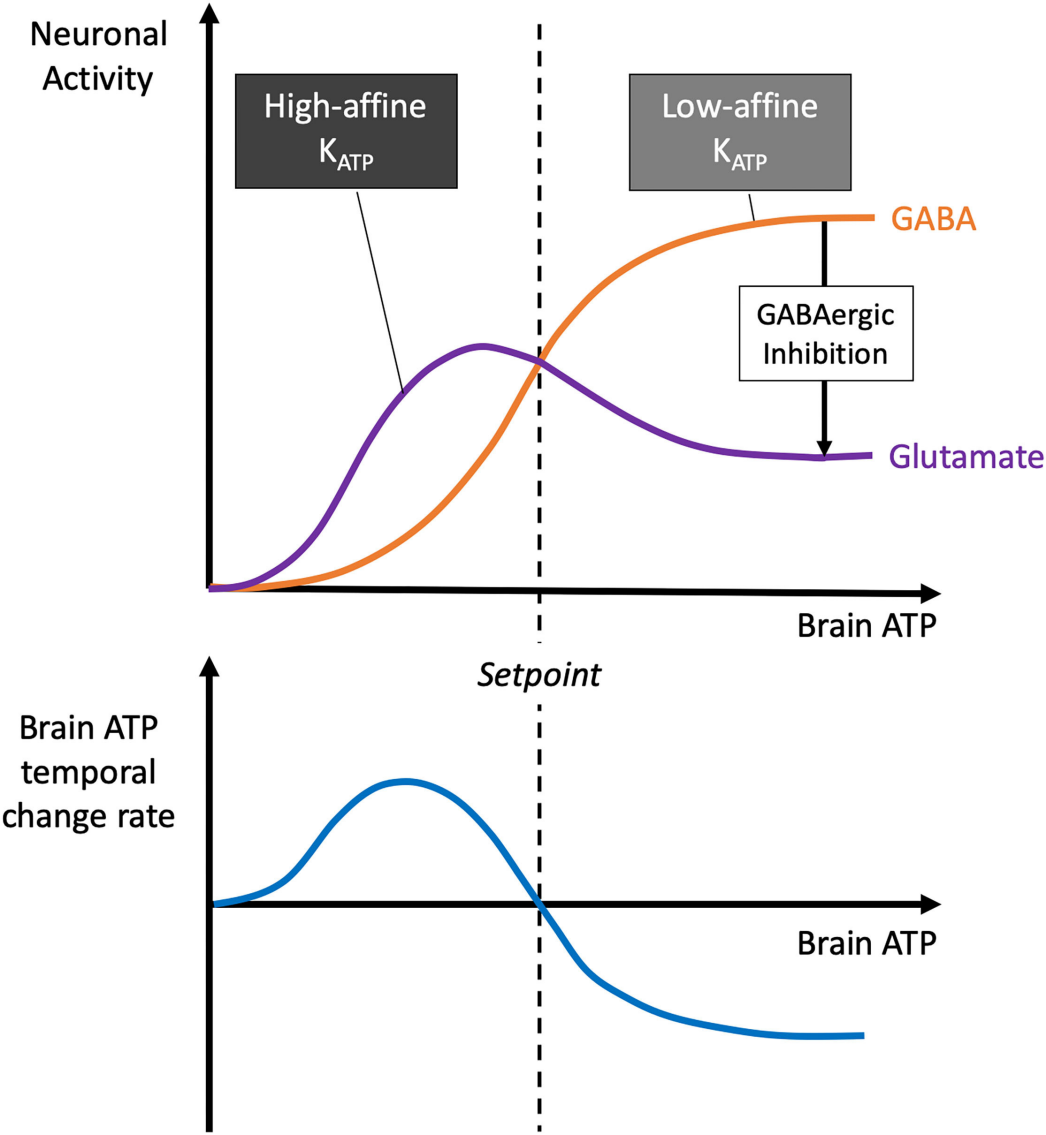
Dependence of brain ATP temporal change rate, glutamatergic and GABAergic neuronal activity on brain ATP. The model shown here was formulated almost 20 years ago (Peters et al., 2004): As brain ATP decreases, presynaptic GABAergic neurons hyperpolarise first due to their low-affinity K_ATP_ channels, followed by postsynaptic glutamatergic neurons with their high-affinity K_ATP_ channels. Since glutamatergic neurons actively pull energy and GABAergic ones do not, a predominance of glutamatergic neurons in the low brain ATP range leads to positive brain ATP temporal change rates, while the predominance of GABAergic neurons in the high brain ATP range leads to negative rates. The brain ATP temporal change rate function $\frac{dATP_{brain}}{dt}$ has a zero where *ATP*_*brain*_ is in equilibrium. This is the setpoint where brain energy homeostasis manifests itself.

The demand and need of glutamatergic and GABAergic neuron populations can be fundamentally changed during a lack or excess of brain energy concentrations. In this respect, the Selfish-Brain theory made detailed predictions many years ago (Peters et al., 2004): These forecasts were based on experimental findings showing that in different brain regions, e.g., cortex, hippocampus, there is a typical constellation where low-affinity ATP-sensitive potassium (K_ATP_) channels are localised on presynaptic GABAergic neurons and high-affinity K_ATP_ channels on post-synaptic glutamatergic neurons (Luhmann and Heinemann, 1992; Ohno-Shosaku et al., 1993; Lee et al., 1996; Jiang and Haddad, 1997) (Figure 5). Latest work also shows the key role of high affinity K_ATP_ channels on pyramidal cells (Karagiannis et al., 2021). The predictions were as follows: When neuronal ATP concentrations fall, this type of multi-site neuronal ensemble leads to biphasic responses. In mild ATP deficiency, low-affinity K_ATP_ channels hyperpolarise GABAergic neurons, thereby disinhibiting post-synaptic glutamatergic neurons and increasing glutamatergic activity (which serves a pull function), while in severe ATP deficiency, high-affinity K_ATP_ channels hyperpolarise glutamatergic neurons, causing glutamatergic activity to cease (which serves a neuroprotective function). Such a theory-predicted biphasic response to falling ATP levels could indeed be confirmed experimentally (Steinkamp et al., 2007). However, in the case we are interested in here, namely the case of an intraneuronal ATP excess, the presynaptic K_ATP_ channels of the GABAergic neurons close completely, so that these neurons are more likely to depolarise. As the presynaptic GABAergic neurons inhibit the postsynaptic glutamatergic neurons, an ATP surplus will shift the glutamatergic/GABAergic balance towards the GABAergic neuron population.

A shift of the glutamatergic/GABAergic balance has two major consequences, one for the cerebral energy budget, the other for cognitive function. The cerebral energy budget is affected in that the glutamatergic neurons, which are the ones involved in the energy demand process, become less active in the presence of surplus energy. As a result, the ensemble of astrocytes, glutamatergic neurons and GABAergic neurons takes up less energy during a cerebral energy surplus. The ATP surplus is thus reduced over time and energy homeostasis is restored. This prediction, based on the Selfish-brain theory, is consistent with the finding from our current systematic review that despite hyperglycaemia brain energy homeostasis can be maintained.

Cognitive function is also affected by a predominance of cerebral GABA tone. Since prevailing GABA-inhibition of glutamatergic neurotransmission limits information processing, we would expect cognitive dysfunction to occur. In fact, cognitive dysfunction has been shown to occur with hyperglycaemia, no matter whether type 1 diabetes or type 2 diabetes (Moheet et al., 2015). In type 2 diabetes, hyperglycaemia was found associated with increased cerebral GABA levels, which went along with cognitive dysfunction (van Bussel et al., 2016; Thielen et al., 2019). STZ diabetes was found to exhibit decreased cerebral glutamate levels and increased cerebral GABA levels (Datusalia and Sharma, 2016), and the animals displayed cognitive dysfunction (Lin et al., 2018). The protection of the cerebral energy budget against hyperglycaemic oversupply thus has its price, namely the impairment of cognitive performance.

Systemic brain-pull function is mainly carried out by the sympathetic nervous system (SNS). The SNS supplies the brain with additional energy when needed. Systemic brain activity may result in the blood containing more substrates (e.g., glucose, ketones, or lactate) for brain use (Hitze et al., 2010; Kubera et al., 2012, 2014) or in more blood flowing to the brain (e.g., due to increased heart rate, which increases cardiac output) (Jones et al., 2011).

More substrates for brain use can be provided by different mechanisms. In the VMH, energy sensors are positioned to detect a decrease in cerebral ATP concentrations. Specifically, VMH neurons are targeted by GABAergic presynaptic nerve terminals, the latter being equipped with K_ATP_ channels (Chan et al., 2007). Upon the decline in ATP concentrations in the VMH, the K_ATP_ channels can no longer bind ATP, so that the presynaptic GABAergic nerve terminals become hyperpolarised, thereby disinhibiting the postsynaptic glutamatergic VMH neurons. The postsynaptic VMH neurons, *via* their glutamatergic output, activate the autonomic centres in the brainstem, which in turn increase the tone of the SNS (Tong et al., 2007; Lindberg et al., 2013). SNS activation in turn leads to the release of noradrenaline from the sympathetic nerve endings and adrenaline from the adrenal medulla. In one of the sympathetic target organs, the endocrine pancreas, SNS nerves stimulate the release of glucagon from alpha cells (Havel et al., 1996) and suppress the release of insulin from beta cells (Ahren, 2000).

Some researchers studied the behaviour of the VMH-pancreas axis as a whole. Chan et al. (2007) showed that a decrease in GABA release in the VMH increased plasma adrenaline and glucagon levels. Stanley showed that acute activation of specific VMH neurons increases plasma glucose and glucagon, but suppresses insulin levels (Stanley et al., 2016). And Meek and colleagues showed that the activation of certain VMH neurons increases blood glucose without increasing insulin concentrations (Meek et al., 2016).

According to the supply chain principle “when push increases, pull relaxes,” the brain-pull is expected to be turned down. Indeed, Chan et al. showed in another paper that STZ diabetic rats exhibited increased GABAergic inhibition in the VMH resulting in blunted glucagon and adrenalin responses (Chan et al., 2011). Furthermore, the researchers showed that when GABA tone in the VMH of STZ-induced diabetic animals was lowered either pharmacologically or genetically, they were able to restore the absent glucagon response and also normalise the impaired adrenalin response (Chan et al., 2011). This study in STZ rats clearly showed that glucagon is in principle present in pancreatic alpha cells, but is only suppressed by output from the VMH. The brain, by down-regulating SNS activity, can suppress glucagon secretion, thus limiting further hepatic glucose production.

In T1DM, insulin secretion can no longer be controlled by the brain, as the irreversible damage to the beta cells leaves no insulin at all. Thus, from the onset of type 1 diabetes, an important brain-pull mechanism for controlling glucose energy flows within the organism gets lost, namely cerebral insulin suppression (CIS) (Woods and Porte, 1974; Ahren, 2000; Hitze et al., 2010). Normally, as mentioned earlier, the brain can suppress insulin secretion via sympathetic efferents when it needs energy (Ahren, 2000). This suppression prevents muscle and adipose tissues from taking up glucose via insulin-dependent pathways (Shepherd and Kahn, 1999) and instead makes the circulating glucose available to the brain via insulin-independent pathways (Deng et al., 2014). For the brain needs virtually no insulin to take up glucose (Hom et al., 1984; Hasselbalch et al., 1999; Seaquist et al., 2001). But if insulin is completely absent, the neuroendocrine mechanisms can no longer let insulin levels rise, so they cannot make surplus blood glucose be stored in muscle and fat tissue. The impact of this inability is reflected in the massive body mass change of −30.7 ± 13.3% (compared to controls) that was evident in this systematic review (Table 1). Such an ineffectiveness of CIS in T1DM means that the other brain-pull mechanisms that act through the sympathetic nervous system have to be turned down in order to protect the brain against oversupply. For example, a downregulated sympathetic nervous system could contribute to the reductions in heart rate (Howarth et al., 2005), circadian variation in heart rate and pulse pressure (Hicks et al., 1998) observed in type 1 diabetes. In all, downregulation of brain-pull mechanisms (shifted glutamatergic/GABAergic balance, reduced SNS activity and glucagon secretion) can help maintain cerebral energy homeostasis despite increased blood-push (hyperglycaemia).

The dataset found in our current systematic review violates the predictions of the gluco-lipostatic theory and its variants, which view the brain as purely passively supplied. From the perspective of these theories, experimental T1DM is expected to affect the energy content of the blood and brain in the same order of magnitude – which was not the case. This is the third time that the gluco-lipostatic theory and its variants have failed the test:

- First, these long-held theories could not predict that a *distal disruption* of cerebral energy supply (caloric restriction) would leave brain mass (energy) virtually unaffected (Sprengell et al., 2021a).
- Second, these theories could not predict that a *proximal disruption* of brain energy supply (cerebral artery occlusion) would have a clinically relevant effect on peripheral energy metabolism, i.e., an increase in systemic blood glucose (Sprengell et al., 2021b).
- Third, as shown in the current paper, these theories could not predict that hyperglycaemic oversupply of the brain that occurs with *peripheral disruption* of energy storage (T1DM) would leave brain mass (energy) virtually unaffected.

These tests systematically examined the three possible disruptions within the cerebral supply chain (Figure 1). We pre-registered all three tests at PROSPERO before we started any of our systematic literature searches. The evidence obtained from this testing may give reason to update the basic tenets of energy metabolism. Updating the long-held theory could replace a brain that is only passively supplied with a brain that independently regulates its energy concentrations and takes a primary position in a hierarchically organised energy metabolism. The latter proposition represents the axiom of the Selfish-Brain theory (Peters et al., 2002).

What could be the clinical significance of updating the basic beliefs about energy metabolism? Of course, the reviewed cases of caloric restriction, cerebral artery occlusion, and type 1 diabetes represent only a very limited group of metabolic states. In contrast, stress, sleep, obesity, and type 2 diabetes mellitus are much more common and have greater overall clinical relevance. Mental stress increases global brain glucose uptake and deep sleep decreases it, indicating that brain energy metabolism is not a constant (Maquet et al., 1990; Madsen et al., 1995). Most research on obesity and type 2 diabetes still relies on the gluco-lipostatic theory and its variants (Schwartz et al., 2017). Given that these gluco-lipostatic theories exhibit explanatory abnormalities, as demonstrated by the three-part series of systematic reviews completed here, the question arises whether these shortcomings also affect mainstream explanatory models for obesity and type 2 diabetes. Do these models overlook an important influence of the *brain's energy demand and need* on peripheral energy metabolism? Therefore, in the next research step, it should be questioned whether the long-held explanatory models for obesity and type 2 diabetes still offer the best explanation.

## Data Availability Statement

The data analysed in this study is subject to the following licences/restrictions: systematic review. Requests to access these datasets should be directed to achim.peters@uksh.de.

## Author Contributions

MS developed the search strategies that BK and AP approved. MS screened the article titles or abstracts against the inclusion and exclusion criteria. BK checked this step, and disagreements were resolved where necessary by consulting AP. MS and BK independently analysed the full text, and disagreements were resolved where necessary by consultation with AP. MS extracted the data, which BK and AP independently checked. MS assessed the risk of bias, which BK and AP independently checked and approved. BK and AP wrote the manuscript. All authors read and approved the submitted version.

## Conflict of Interest

The authors declare that the research was conducted in the absence of any commercial or financial relationships that could be construed as a potential conflict of interest.

## Publisher's Note

All claims expressed in this article are solely those of the authors and do not necessarily represent those of their affiliated organizations, or those of the publisher, the editors and the reviewers. Any product that may be evaluated in this article, or claim that may be made by its manufacturer, is not guaranteed or endorsed by the publisher.
