# Supplementary material for: Brain Mass (Energy) Resistant to Hyperglycaemic Oversupply: A Systematic Review
Source: Front Neurosci. 2021 Nov 4;15:740502. doi: 10.3389/fnins.2021.740502 (PMC8600366; doi:10.3389/fnins.2021.740502)
Supplement: Supplementary file 1 [file Table_1.DOCX]

**SUPPLEMENTARY MATERIAL**

**A. Search strategy MEDLINE via PubMED**

#1 ((Diabetes Mellitus[MH] OR diabetic[TI] OR diabetes[TI]) AND

(Streptozocin[MH] OR Pancreatectomy[MH] OR Streptozocin[TIAB] OR streptozotocin[TIAB] OR STZ[TIAB] OR alloxan[TIAB] OR Pancreatectomy[TIAB] OR pancreatectomized[TIAB] OR depancreatized[TIAB] OR (experimental[TI] AND induced[TI]) OR (chemically[TI] AND induced[TI])))

#2 **((Brain[TIAB] OR cerebral[TIAB] OR “central nervous system”[TIAB] OR CNS[TIAB]) AND** (Energy Metabolism[MH] OR "Adenosine Triphosphate"[MH] OR ATP[TIAB] OR „Adenosine triphosphate“[TIAB] OR “high energy phosphate”[TIAB] OR “high energy phosphates”[TIAB] OR “energy state”[TIAB] OR “phosphorus magnetic resonance spectroscopy”[TIAB] OR “p magnetic resonance spectroscopy”[TIAB] OR “phosphorus MRS”[TIAB] OR “p MRS”[TIAB] OR mass[TIAB] OR weight[TIAB] OR size[TIAB] OR volume[TIAB] OR “magnetic resonance imaging”[TIAB]) OR “organ mass”[TIAB] OR “organ size”[TIAB])

#3 Animal Experimentation[MH] OR Animals[MH] OR Random Allocation[MH] OR randomized controlled trial[PT] OR clinical trial, veterinary[PT] OR randomization[ALL] OR experiment[ALL] OR intervention*[TIAB] OR groups[TIAB] OR randomly[TIAB] OR randomized[TIAB] OR trial[TIAB] OR “animal study”[TIAB] OR “control group”[TIAB]

#4 #1 AND #2 AND #3 NOT review[ALL] NOT patients[TI] NOT women[TIAB] NOT men[TIAB] NOT cohort[TIAB] NOT insipidus[TI] NOT “type 2”[TI] NOT “type-2”[TI] NOT “type II”[TI]

**B. Search Strategy Biosis Previews via Biosis Citation Index**

#1 TI=((“diabetic” OR “diabetes”) AND (“streptozocin” OR “streptozotocin” OR “STZ” OR “alloxan” OR “pancreatectomy” OR “pancreatectomized” OR “depancreatized” OR (“experimental” AND “induced”) OR (“chemically” AND “induced”)))

#2 TS**=(((“brain” OR “cerebral” OR “central nervous system” OR “CNS”) AND** (“energy metabolism” OR "adenosine triphosphate” OR “adenosine triphosphates” OR “ATP” OR “high energy phosphate” OR “high energy phosphates” OR “energy state” OR “phosphorus magnetic resonance spectroscopy” OR “p magnetic resonance spectroscopy” OR “phosphorus MRS” OR “p MRS” OR “mass” OR “weight” OR “size” OR “volume” OR “magnetic resonance imaging”)) OR “organ mass” OR “organ size”)

#3 TS=(“animal experimentation” OR “animals” OR “random allocation” OR “randomized controlled trial” OR “randomization” OR “experiment” OR “intervention” OR “interventions” OR “groups” OR “randomly” OR “randomized” OR “trial” OR “animal study” OR “control group”)

#4 #1 AND #2 AND #3 NOT TI=(“review” OR “patients” OR “women” OR “men” OR “cohort” OR “insipidus” OR “type 2” OR “type-2” OR “type II”)

**C. Anaesthesia before ATP measurement**

**Supplementary figure 1: Anaesthesia before ATP measurement and the relationship between blood and brain outcomes.** The plot shows STZ- or alloxan-induced percentage changes in blood glucose levels (compared to controls) and in ATP levels (compared to controls); anaesthesia before ATP measurement (red symbols), no anaesthesia before ATP measurement (green symbols). Anaesthesia before ATP measurement does not seem to confound the relationship between blood and brain outcomes.
